# Treatment of Glucocorticoids Inhibited Early Immune Responses and Impaired Cardiac Repair in Adult Zebrafish

**DOI:** 10.1371/journal.pone.0066613

**Published:** 2013-06-21

**Authors:** Wei-Chang Huang, Chung-Chi Yang, I-Hui Chen, Yu-Min Lawrence Liu, Shing-Jyh Chang, Yung-Jen Chuang

**Affiliations:** 1 Department of Medical Science & Institute of Bioinformatics and Structural Biology, National Tsing Hua University, Hsinchu, Taiwan; 2 Division of Cardiology, Taoyuan Armed Forces General Hospital, Taoyuan, Taiwan; 3 Division of Cardiology, Department of Internal Medicine, Mackay Memorial Hospital Hsinchu Branch, Hsinchu, Taiwan; 4 Department of Obstetrics and Gynecology, Mackay Memorial Hospital Hsinchu Branch, Hsinchu, Taiwan; Institute of Clinical Medicine, National Cheng Kung University , Taiwan

## Abstract

Myocardial injury, such as myocardial infarction (MI), can lead to drastic heart damage. Zebrafish have the extraordinary ability to regenerate their heart after a severe injury. Upon ventricle resection, fibrin clots seal the wound and serve as a matrix for recruiting myeloid-derived phagocytes. Accumulated neutrophils and macrophages not only reduce the risk of infection but also secrete cytokines and growth factors to promote tissue repair. However, the underlying cellular and molecular mechanisms for how immune responses are regulated during the early stages of cardiac repair are still unclear. We investigated the role and programming of early immune responses during zebrafish heart regeneration. We found that zebrafish treated with an anti-inflammatory glucocorticoid had significantly reduced heart regenerative capacities, consistent with findings in other higher vertebrates. Moreover, inhibiting the inflammatory response led to excessive collagen deposition. A microarray approach was used to assess the differential expression profiles between zebrafish hearts with normal or impaired healing. Combining cytokine profiling and immune-staining, our data revealed that impaired heart regeneration could be due to reduced phagocyte recruitment, leading to diminished angiogenesis and cell proliferation post-cardiac injury. Despite their robust regenerative ability, our study revealed that glucocorticoid treatment could effectively hinder cardiac repair in adult zebrafish by interfering with the inflammatory response. Our findings may help to clarify the initiation of cardiac repair, which could be used to develop a therapeutic intervention that may enhance cardiac repair in humans to compensate for the loss of cardiomyocytes after an MI.

## Introduction

Cardiovascular diseases have long been the leading cause of morbidity and mortality worldwide [1], in which myocardial infarction (MI) is associated with the highest risk of death and complications [2]. After a MI episode, the blood supply to the affected region is disrupted, which causes cardiomyocyte death in the infarcted area. In patients who survive the MI, fibrotic tissue replaces the damaged myocardium [3], resulting in reduced systolic function. In addition, fibrotic scar tissue disrupts previously synchronized ventricular contraction, leading to pathological cardiac remodeling, heart failure and eventual death. Thus, developing therapeutic approaches to repair the damaged heart is a major challenge for researchers and physicians alike [4].

Different from the four-chambered human heart, zebrafish (*Danio rerio*) heart has a simply two-chambered structure and is composed of one atrium and one ventricle. Despite the anatomical difference, numerous studies have shown that the embryonic morphogenesis, essential genes expression programming, and electrical activity of the human and zebrafish hearts are highly conserved, making zebrafish a suitable model organism to study cardiac development and related human diseases [5]. Unlike mammals, zebrafish have an astounding ability to regenerate many vital organs, including the heart [6] and central nervous system [7], which are difficult for fully grown mammals to repair after traumatic injuries [8]. Numerous studies have demonstrated that the adult zebrafish can fully repair its damaged heart from up to a 20% ventricular resection within two months. Massive cell proliferation occurs at the zebrafish injury site can overcome scar formation and allow the cardiac muscle to regrow fully [6]. Intriguingly, recent studies have suggested that adult mammals actually retain some ability to repair a damaged heart. According to an isotope tracking study by Bergmann *et al.*, more than 50% of cardiomyocytes in humans have been estimated to be replaced after birth [9]. A murine genetic fate tracking study also demonstrated that the mammalian myocardium can partially be regenerated after cardiac injury [10]. However, such regenerative ability cannot compensate the loss of cardiomyocytes in conditions like MI occurred in adult mammals.

Numerous wound healing studies have shown that inflammatory responses are highly associated with tissue repair. For example, glucocorticoid-treated mice failed to regenerate the wounded skin, which is accompanied by a significant down-regulation of pro-inflammatory cytokines [11]. Furthermore, glucocorticoid administration was associated with delayed clearing of dead cardiomyocytes in mice [12], indicating that the inflammatory process is tightly intertwined with post-infarction cardiac repair. In zebrafish, administration of glucocorticoids like beclomethasone also resulted in failed fin regeneration; however, it has been suggested that inflammatory response may not be necessary for accomplishing regeneration in the fin model [13]. Recently, in a traumatic brain injury model of zebrafish, Kyritsis *et al.* demonstrated that acute inflammation is necessary and sufficient for activating injury-induced molecular programs, promoting the proliferation of neural progenitors and subsequent neurogenesis [14]. Together, these studies have suggested that the initiation and control of tissue repair are highly associated with early immune responses in either the mouse or zebrafish, which may share a well-conserved mechanism for initiating cardiac repair among the vertebrates. However, the detailed correlation between acute immune regulation and the subsequent cardiac repair still remain unclear at this time.

In this study, we aimed to characterize the early inflammatory responses (within 72 hours post injury) during zebrafish cardiac repair. We found that zebrafish treated with an anti-inflammatory steroid had a significant reduction in cardiac regeneration capability. A microarray approach was used to evaluate the differential expression of the wound healing transcriptome in the hearts of normal and immunosuppressed zebrafish to provide an overall assessment of the genes that would be important for triggering cardiac repair during the early inflammation phase. Using transgenic labeling and immunostaining assays, we further demonstrated that impaired cardiac repair in zebrafish could be related to diminished phagocytes recruitment, angiogenesis and cell proliferation. Furthermore, inhibition of the immune response led to excess collagen deposition in the wound, supporting the prevailing hypothesis that scar formation opposes regeneration during cardiac repair [15]. Our findings demonstrate that the zebrafish cardiac repair capability can be controlled by manipulating its immune response.

## Results

### Elevation of Pro-inflammatory Cytokines at the Early Stage of Cardiac Repair in Adult Zebrafish

**Figure 1:**
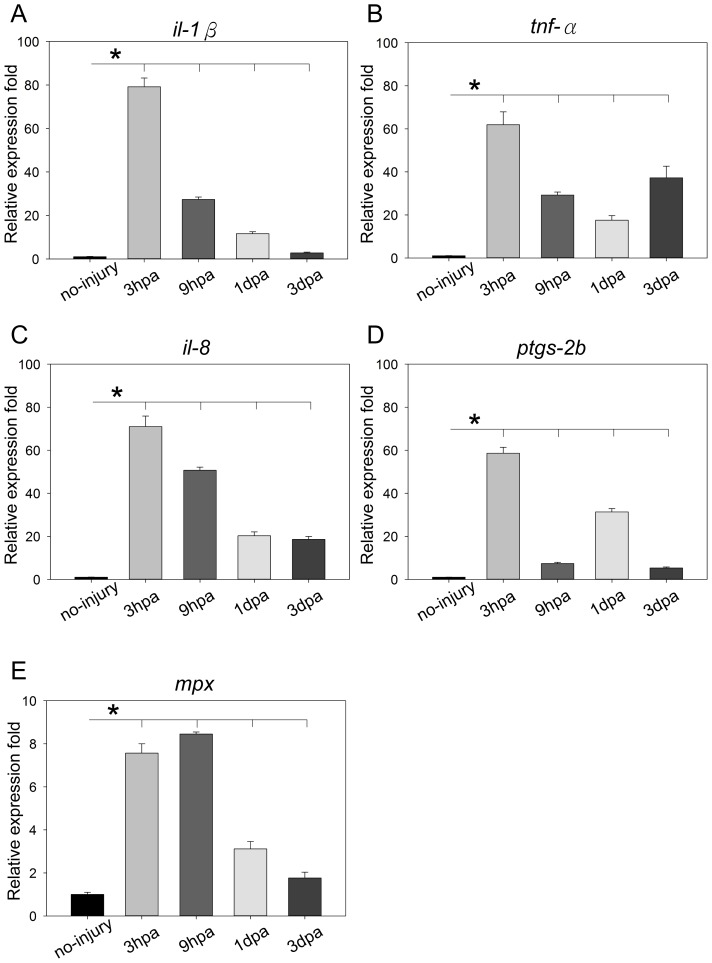
Elevation of pro-inflammatory cytokines during early cardiac repair. Whole hearts were harvested at different time intervals after ventricular resection (no-injury, 3 hpa, 9 hpa, 1 dpa, and 3 dpa). RT-qPCR was conducted using gene-specific primers for *il-1β, tnf-α, il-8, ptgs-2b* and *mpx*. The relative expression level was normalized to the no-injury control group. (A–E) Expression levels were compared to control groups. Strong and early elevation of pro-inflammatory cytokines could be detected early after cardiac injury (n = 3). The data represent the mean± SEM. *indicates p value<0.05.

To investigate the role of inflammation at the early phase of zebrafish cardiac repair, we examined the expression profiles of five inflammatory marker genes, *il-1β*, *tnf-α*, *il-8*, *prostaglandin-endoperoxide synthase 2b (ptgs-2b)* and *myeloid-specific peroxidase (mpx)* within 3 dpa (Fig. 1). IL-1β and TNF-α are well-known pro-inflammatory cytokines that act as essential mediators to amplify inflammatory responses [11]. During the early stages of cardiac repair, a rapid and substantial elevation of both *il-1β* and *tnf-α* transcription could be detected at 3 hours post amputation (hpa). The relative expression folds of *il-1β* were: 79.2-fold at 3 hpa, 27.3-fold at 9 hpa, 11.6-fold at 1 dpa and dropped to 2.8-fold at 3 dpa (Fig. 1A). For *tnf-α* mRNA expression: 61.9-fold at 3 hpa, 29.2-fold at 9 hpa and 17.5-fold at 1 dpa. Interestingly, *tnf-α* mRNA expression was up-regulated again to 37.2-fold, which indicates a second wave of *tnf-α* induction has occurred in the early stages of cardiac repair (Fig. 1B). IL-8 is known to serve as a key chemokine for phagocytes recruitment during wound healing and also as a pro-angiogenic factor to promote vascular remodeling [16]. We observed a significant elevation of *il-8* mRNA expression during the early stages of cardiac repair, with an expression profile less than but comparable to *il-1β.* The relative expression folds of *il-8* were: 71.0-fold at 3 hpa, 50.7-fold at 9 hpa, 20.3-fold at 1 dpa, and 18.6-fold at 3 dpa (Fig. 1C). PTGS-2b, also known as COX-2b, is an enzyme responsible for the synthesis of important inflammatory mediators called prostanoids [17]. The relative expression folds of *ptgs-2b* were: 58.6-fold at 3 hpa, 7.4-fold at 9 hpa, 31.3-fold at 1 dpa and decreased to 5.3-fold at 3 dpa (Fig. 1D), which indicating a second but transient burst of *ptgs-2b* elevation at the early stages of cardiac repair. Myeloid-derived phagocytes recruitment has been observed during cardiac repair [18]; MPX, which is a lysosomal enzyme expressed by neutrophils and commonly denoted as an indicator of inflammation [19]. The relative expression folds of *mpx* were: 7.6-fold at 3 hpa, 8.5-fold at 9 hpa, 3.1-fold at 1 dpa and 1.8-fold at 3 dpa. The expression profile of *mpx* in the damaged heart suggests that, similar to the findings in mammals, neutrophils can be recruited to the site of injury and be activated quickly after traumatic cardiac injury in adult zebrafish (Fig. 1E).

### Inflammation was Required to Complete Cardiac Repair in Adult Zebrafish

**Figure 2:**
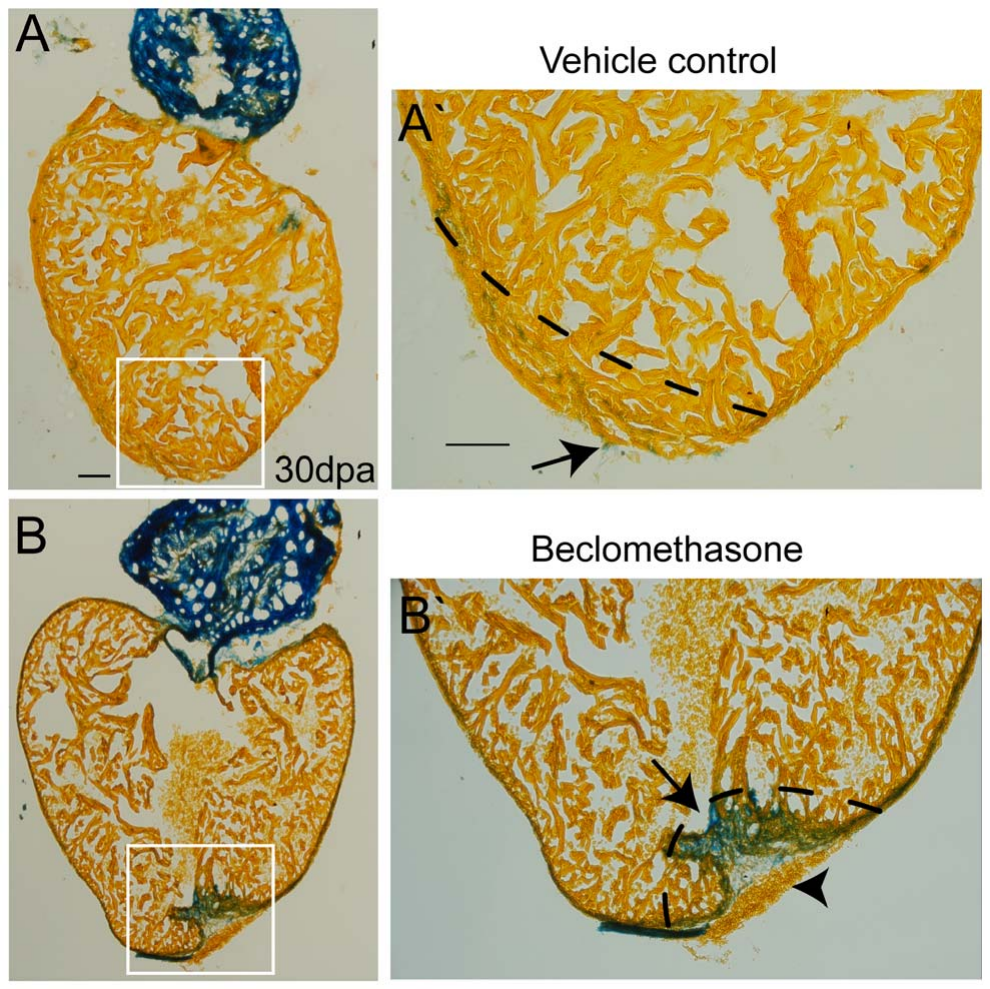
Treatment with beclomethasone caused impaired cardiac repair in zebrafish. Objects were sacrificed at 30 days post ventricular resection; heart sections were stained with aniline blue to distinguish scar tissue from normal tissue. Blue: scar tissue; Orange: normal tissue. (A, A’) In the vehicle control group, the zebrafish regenerated the injured hearts perfectly 1 month after injury. Only a few scar tissue (arrow) was detected in the wound (n = 17). (B, B’) Exposure to beclomethasone (0.25 µM) hindered the cardiac repair process. A large amount of scar tissue (arrow) was found in the wound instead of renewed cardiomyocytes. Meanwhile, obvious blood clots remained, which could be detected (arrow head) (n = 14, scale bar = 100 µm). The dashed lines indicate the approximate amputation plane.

The acute elevation of pro-inflammatory cytokines implied they are important during the early phase of zebrafish cardiac repair. To investigate the role of inflammatory responses during cardiac repair, we treated the zebrafish with the anti-inflammatory glucocorticoid, beclomethasone, for subsequent assays. We found exposure to beclomethasone (0.25 µM) significantly impaired cardiac repair in zebrafish. The aniline blue staining was used to visualize substantial collagen deposition (i.e., scar tissue) in the concave injury site, while a patch of blood clots remained to protect and cover the entire lesion (Fig. 2B and 2B’). In contract, we found that the zebrafish could regenerate from the ventricular amputation by 1 month in the vehicle control group, and only a few traces of collagen were detected (Fig. 2A and 2A’).

To quantify and compare the amount of scar tissue in the recovering wound, we calculated the percentage of scar tissue compared to the entire ventricle. Only 0.42% of the scar tissue was detected in the ventricles of the vehicle control group at 30 dpa. In contrast, approximately 4.23% of the scar tissue still remained in the beclomethasone-treated group, which was not yet replaced by myocardium after 1 month. Our results revealed that scar removal was impaired by beclomethasone treatment in adult zebrafish after ventricular amputation.

Since glucocorticoid steroid treatment has been shown to exert wide impacts on physiology activities other than anti-inflammation, a second drug was used to confirm the essential role of inflammation during zebrafish cardiac repair. In this assay, the NSAID (non-steroidal anti-inflammatory drug) ibuprofen was used to repress inflammation during cardiac repair. Under ibuprofen (0.25 µM) treatment, we observed substantial amount of residual scar tissues and cellular debris within the wound at 1 month post injury (Fig. S1). The results are consistent with the beclomethasone-induced effects. Moreover, we examined and compared levels of the inflammatory marker gene transcripts, *il-1β*, *tnf-α* and *ptgs-2b*, under beclomethasone and ibuprofen treatments after ventricular resection. As expected, both drugs effectively repressed the acute inflammatory responses after cardiac injury at 16 hpa; due to the nature of NSAID, ibuprofen showed a more specific inhibition on *ptgs-2b* as compared with the beclomethasone (Fig. S2). These data suggested that suppression of the acute immune responses during zebrafish cardiac repair would block its inherent regenerative ability.

### Global Gene Expression Analysis Identified Significant Genes Down-regulated by Beclomethasone in Impaired Healing Hearts

We had learned that beclomethasone treatment impaired cardiac repair in zebrafish significantly. To assess the overall differential transcript expression in the initial phase of cardiac repair, we compared the overall gene expression profiles of hearts sampled under three conditions: no-injury, 1dpa-vehicle (i.e., normal healing), and 1dpa-beclomethasone (i.e., impaired healing), using the Agilent zebrafish specific 60-mer oligonucleotide microarray.

Since treatment of beclomethasone had deleterious effects on zebrafish cardiac repair, we mainly focus on the transcripts that were originally elevated after injury while repressed under beclomethasone treatment. After normalization, we identified 2857 *D. rerio* transcript probes that were over-expressed by ≥ 3-fold in the 1dpa-vehicle group compared to the no-injury group. We next identified 4064 transcript probes that were down-regulated by ≥ 3-fold in the 1dpa-beclomethasone group compared to the 1dpa-vehicle group (Fig. S3). From cross-comparison, we obtained a list of 2288 significant *D. rerio* transcript probes that were originally elevated after injury, yet depressed under beclomethasone treatment in post-cardiac injury hearts (Fig. S3 and Dataset S1). The list of 2288 differentially expressed entities was then processed by gene ontology analysis using the PANTHER classification system. We only kept the hits that passed the stringency with a corrected *p* value of less than 0.05.

Based on the gene ontology analysis results, we found that beclomethasone treatment expectedly impaired immune system processes, including an immune response and macrophage activation. These data serve to validate the known immunosuppressive function of beclomethasone; not surprisingly, the down-regulated expression profiles of the 5 inflammatory marker genes were also detected and validated by the microarray analysis. Eight other vital repair processes were also significantly affected by beclomethasone treatment during cardiac repair, including RNA metabolism, system development, cell communication, signal transduction, cell surface receptor linked signal transduction, mesoderm development, cell-cell signaling, and skeletal system development (Table S1).

### Beclomethasone Reduced the Expression of Key Pro-inflammatory Genes and Phagocyte Recruitment after Cardiac Injury

**Figure 3:**
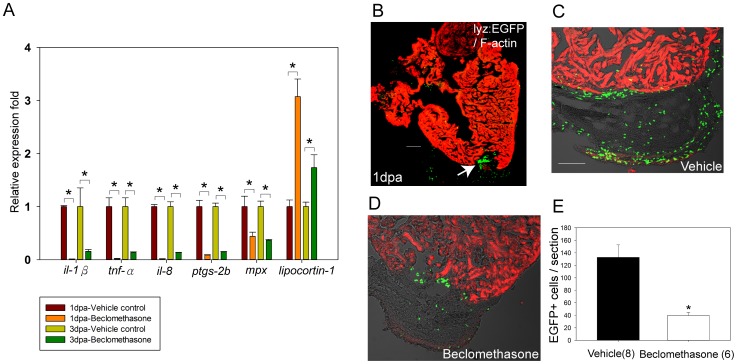
Beclomethasone treatment reduced pro-inflammatory gene expression and phagocyte recruitment after cardiac injury. Whole hearts were harvested at 1 day and 3 days post ventricular resection. RT-qPCR was conducted to quantify the relative fold of pro-inflammatory genes at hearts. (A) Expression of *il-1β*, *tnf-α*, *il-8*, *ptgs-2b*, and *mpx* were all significantly reduced in the beclomethasone-treated group for at least 3 days, while *lipocortin-1* expression was significantly induced after beclomethasone treatment (n = 3). * indicates p<0.05. (B) Immunostaining of hearts after injury. Green: phagocytes (*lyz*-EGFP); red: Phalloidin-546. After cardiac injury, numerous phagocytes accumulated in the wound (arrow). (C, E) In the vehicle control group, average of 133±20 phagocytic cells were counted in the fibrin clots (n = 8). (D, E) In the beclomethasone treated animals, 40±5 cells were counted (n = 6). (E) Treatment with beclomethasone significantly impaired phagocyte recruitment in the injured hearts. The data represent the mean±SEM, *indicates p<0.05, scale bar = 100 µm.

Next, we examined the expression levels of target inflammatory cytokines under beclomethasone treatment by RT-qPCR. We found that expression levels of *il-1β*, *tnf-α*, *il-8*, *ptgs-2b* and *mpx* were all significantly down-regulated in the hearts of beclomethasone-treated group within the first 3 days post injury. Specifically, the normalized expression level of *il-1β* decreased to 1.3% at 1 dpa and 15.8% at 3 dpa of the controls. Similarly, *tnf-α* expression decreased to 1.9% at 1 dpa and 13.5% at 3 dpa; *il-8* expression fell to 1.8% at 1 dpa and 13.3% at 3 dpa; *ptgs-2b* expression decreased to 8.7% at 1 dpa and 14.7% at 3 dpa; and *mpx* expression fell to 44.0% at 1 dpa and 36.4% at 3 dpa of the controls (Fig. 3A).

Previous study suggests that early stage of zebrafish heart regeneration has pre-dominant inflammatory responses, which are similar to the typical wound healing process within the first 3 days [20]. In addition, it has been shown that glucocorticoid-treatment can significantly up-regulate the expression of Lipocortin-1 (also known as Annexin-1), which is a phospholipid-binding protein with phospholipase A2 inhibitory activity [21]. Our data confirmed that *lipocortin-1* mRNA was effectively increased by 3-fold in the injured hearts at 1 dpa, and by 1.5-fold at 3 dap under beclomethasone treatment. This data demonstrated the efficacy of beclomethasone in our assays as compared to the vehicle controls (Fig. 3A).

Since phagocyte recruitment to the injury site is the signature inflammatory response in the initial phase following a cardiac injury, we explored whether the immune cell response was also affected by beclomethasone treatment. For cell tracking, we used the transgenic zebrafish line Tg(*lyz*:EGFP), in which the lysozyme C promoter drives the expression of green fluorescent protein in myeloid-derived phagocytes. Under control conditions, abundant phagocytes were recruited to the wound at 1 day post cardiac injury (Fig. 3B). Clusters of EGFP-labeled phagocytes were found within the blood clots (Fig. 3C). In comparison, beclomethasone treatment resulted in a significant reduction in phagocyte recruitment at 1 dpa (Fig. 3D). The average number of phagocytes accumulating in the wound was calculated, and there were 133±20 and 40±5 phagocytes/section in the control and the beclomethasone-treated groups, respectively. The data translated to a 69.9% reduction in phagocyte recruitment under the influence of beclomethasone (Fig. 3E).

### Angiogenesis was Impaired in the Beclomethasone-treated Zebrafish

From the microarray analysis, specific physiological processes were identified to be inhibited by beclomethasone treatment during cardiac repair in zebrafish. Two processes of interest were angiogenesis and heart development (Table S1). We then surveyed the list of 2288 transcript entities to seek out representative genes involved in angiogenesis and heart development (Dataset S1). We identified *fgfr1a* [22], *vegfaa* [23] and *pcna* [24], which are well-known factors that regulate angiogenesis and cell proliferation. It is noteworthy that previous studies also demonstrated the critical role of *fgfr1* in zebrafish cardiac repair [25]. The rapid up-regulation of these genes after injury implied that cell proliferation and angiogenesis signaling pathways may be linked to the initial phase of cardiac repair; their down-regulation during beclomethasone treatment could therefore cause impaired healing of the damaged heart.

**Figure 4:**
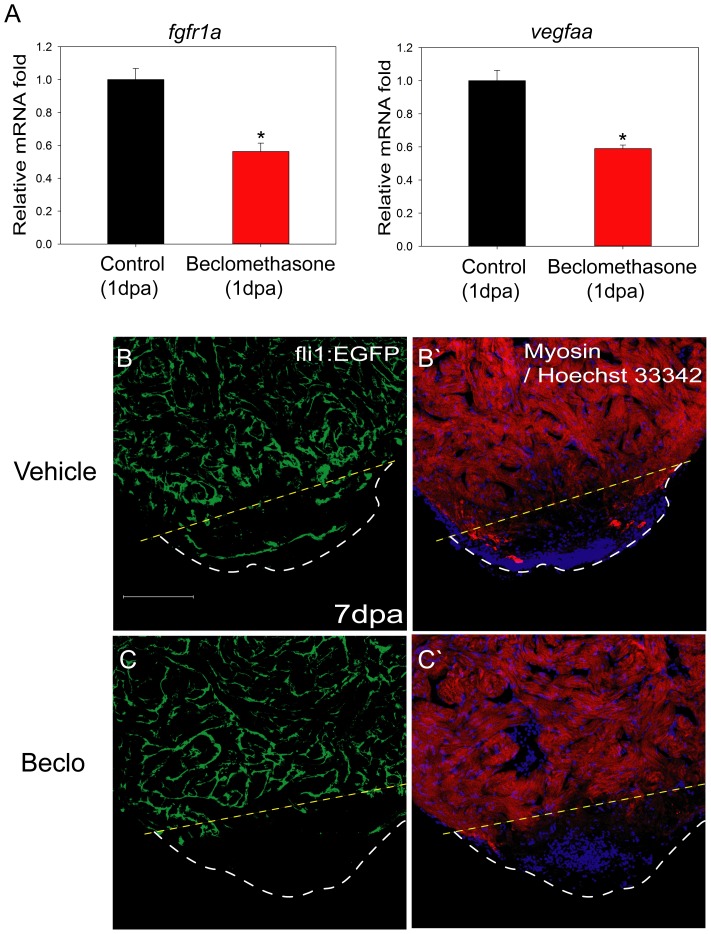
Angiogenesis was hindered in the beclomethasone-treated zebrafish during cardiac repair. We checked pro-angiogenic genes of drug-treated objects at early regeneration and then examined neo-vascularization at 7 dpa. (A) At 1 dpa, mRNA expression of pro-angiogenic genes was reduced after beclomethasone treatment with a 43.7% reduction for *fgfr1a* and 41% for *vegfaa*. The data represent the mean±SEM. *indicates p<0.05. (B, B’) At 7 dpa, there were blood vessels appearing in the clotted areas of the control animals. (C, C’) The beclomethasone-treated animals showed little formation of new blood vessels. The yellow dashed lines indicate the approximate amputation plane; the white dashed lines indicate the outline of the apex. Scale bar = 100 µm.

To verify this, we examined their mRNA expression by RT-qPCR and revealed that under beclomethasone treatment, *fgfr1a* expression would decrease to 56.3±5.1% at 1 dpa; while *vegfaa* expression fell to 59.0±2.1% (Fig. 4A). We presumed that such a reduction in the pro-angiogenic transcripts in the early stages of cardiac repair would lead to obstruction of subsequent neovascularization. To further prove this, transgenic Tg(*fli1a*:EGFP) zebrafish with blood vessels endogenously labeled with green fluorescence were used. Under normal healing conditions, recovered or newly formed blood vessels can be observed at 7 days post injury (Fig. 4B); while in beclomethasone-treated impaired healing hearts, only a few traces of angiogenic vessels could be found (Fig. 4C). For quantitative analysis, the total neovascularization was significantly reduced in the impaired healing group. We detected 3.46±0.37% neovascularization (blood vessels area/wound area) in the normal healing hearts, and a significant reduction to 0.51±0.12% neovascularization in the impaired healing group, which translates to a striking 85.26% reduction in angiogenesis by the beclomethasone treatment.

### Cell Proliferation was also Impeded in Beclomethasone-treated Zebrafish

In adult zebrafish, the restoration of the damaged or missing cardiac muscle after injury is contributed primarily by residential cardiomyocyte proliferation [26]. To assess whether impaired cardiac repair caused by beclomethasone treatment would also result in diminished cardiomyocytes proliferation, we examined *pcna* and *nkx2.5* expression in the control and beclomethasone-treated hearts post cardiac injury.

**Figure 5:**
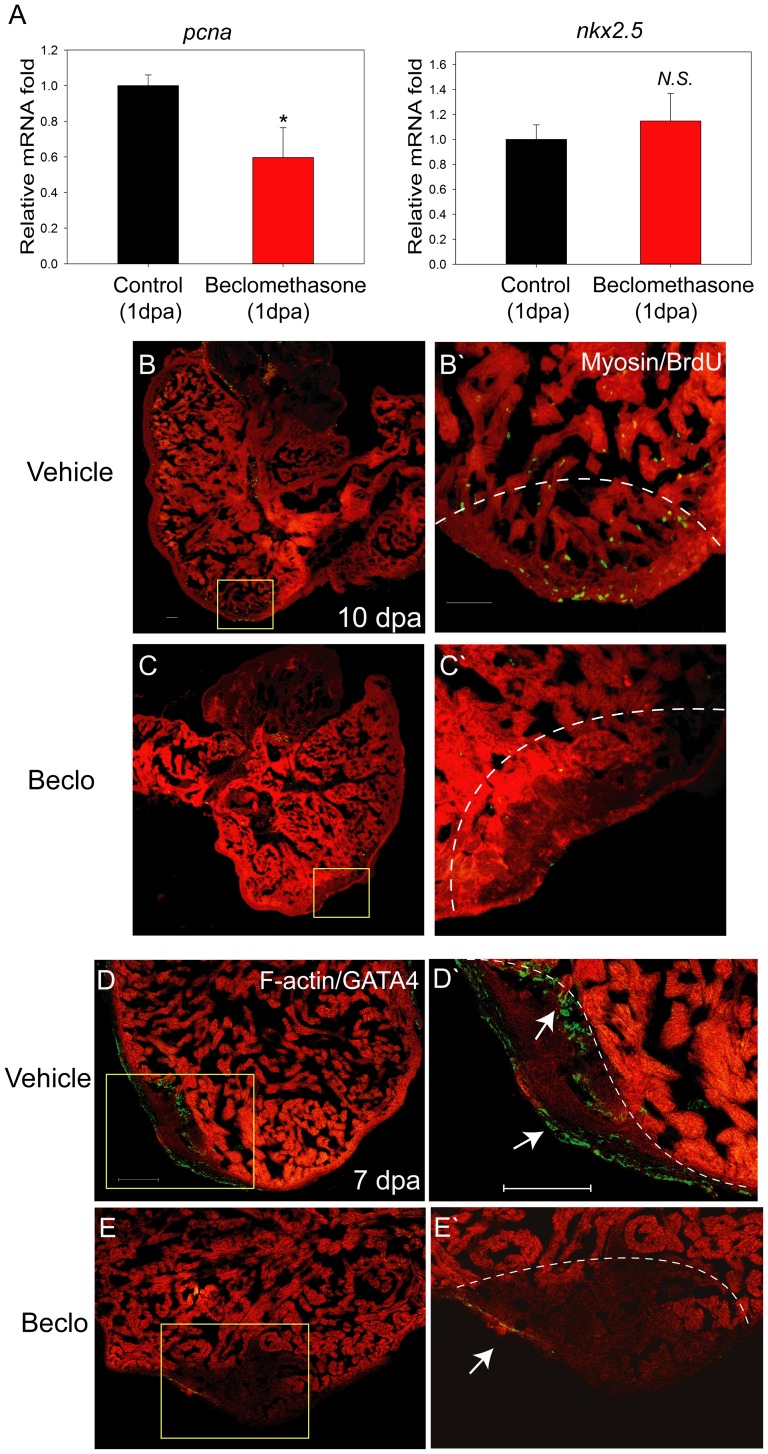
Cell proliferation was hindered in the beclomethasone-treated zebrafish during cardiac repair. Cell proliferation was examined in early phase of regeneration. (A) At 1 dpa, mRNA expression of proliferation marker gene, *pcna,* was reduced after beclomethasone treatment (40.3% reduction). In contract, the expression of the pre-cardiac gene, *nkx2.5*, showed no significant difference between the two groups. The data represent the mean±SEM. *indicates p<0.05. N.S. indicates no significant difference. (B, B’) In the control animals, numerous proliferating BrdU^+^ cells could be detected in the wound (n = 6). The dashed lines indicate the approximate amputation plane. (C, C’) The beclomethasone-treated animals showed little cell proliferation activities (n = 7, scale bar = 50 µm). (D, D’) In control groups, GATA4^+^ regenerating cells appeared near the injury site at 7 dpa. (arrow, n = 5) (E, E’) Beclomethasone treatment significantly reduced the GATA4^+^ regenerating cells during cardiac repair (arrow, n = 5, scale bar = 100 µm). The dashed lines indicate the approximate amputation plane.

By RT-qPCR, we verified that expression of the cell proliferation marker *pcna* significantly decreased to 59.7±16.7% after beclomethasone treatment at 1 dpa (Figure 5A). We next checked the expression of the early pre-cardiac gene *nkx2.5*, whose expression pattern was used to identify the heart field and shown to initiate cardiomyocyte differentiation during development [27]. Interestingly, we observed no significant difference in the expression level of *nkx2.5* between the vehicle control (1.0±0.1-fold in mRNA expression) and the beclomethasone-treated (1.1±0.2-fold in mRNA expression) groups (Fig. 5A). Our data imply that beclomethasone treatment did not alter the cell fate decision of early cardiac precursor cells in the post-injury heart. Instead, beclomethasone impaired overall cardiac cell proliferation in the initial phase of cardiac repair.

Because previous studies have suggested that cell proliferation occurs at 7 dpa or even earlier [6], we investigated the beclomethasone effect on the cell cycle entry using the BrdU assay. We found numerous BrdU-labeled cells in the wound at 10 dpa, suggesting robust cell proliferation activity (Fig. 5B and 5B’), with slightly more proliferating cells located on the recovering tip of the ventricle (Fig. 5B’). However, treatment with beclomethasone resulted in a drastic reduction of cell proliferation activity, accompanied by an obvious hollow cavity found at the injury site (Fig. 5C and 5C’). Quantitative analysis further revealed that there were 52±6 BrdU-positive cells in the wound for the normal healing group, whereas there were only 14±2 cells for the impaired healing group, demonstrating a prominent 73.08% reduction in cell proliferation activity.

Previous study suggested that sub-epicardium derived GATA4 positive cells contributed majorly to the regenerating zebrafish hearts [28]. To check whether GATA4 positive regenerating cells were affected under beclomethasone treatment, we applied immune-staining assay to investigate the expression of GATA4 in zebrafish hearts. In the control objects, we found profound numbers of GATA4 positive cells appearing around the injury site at 7 dpa (Fig. 5D and 5D’). In contrast, the beclomethasone treated hearts had relatively few GATA4 positive cells at the wound (Fig. 5E and 5E’). This data implied the anti-inflammatory effect of the beclomethasone may cause a significant reduction of regenerating cells during zebrafish cardiac repair.

### Wnt16 was Inhibited by Beclomethasone in the Early Phase of Zebrafish Cardiac Repair

**Figure 6:**
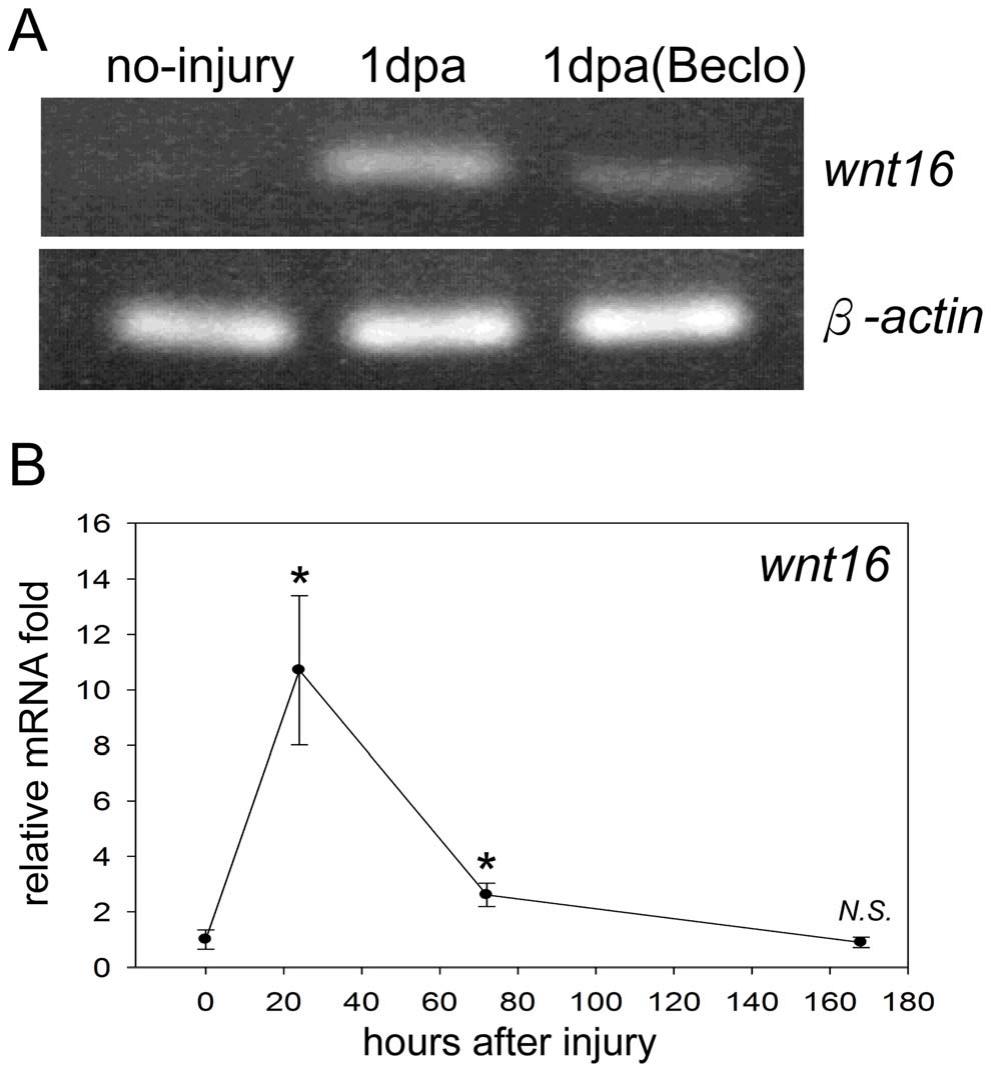
*wnt16* was inhibited by beclomethasone in the early phase of zebrafish cardiac repair. RT-PCR assays revealed the normal injury response of *wnt16* expression patterns, and confirmed the inhibitory effect of beclomethasone on *wnt16* mRNA expression in zebrafish heart. We then checked the mRNA expression of *wnt16* before ventricular resection and at 1 dpa, 3 dpa and 7 dpa by RT-qPCR. (A) Zebrafish *wnt16* mRNA was elevated at 1 dpa after injury. In contrast, its expression was repressed by beclomethasone treatment at 1 dpa. (B) During zebrafish cardiac repair, *wnt16* transcript was highly elevated at 1 dpa. Its level was diminished by 3 dpa, and gradually reduced to the basal level by 7 dpa. (n = 3) The data represent the mean±SEM. *indicates p<0.05. N.S. indicates no significant difference compared to no-injury control.

The Wnt signaling pathway is required during development and regeneration [29], [30]. A recent zebrafish study demonstrates the role of *wnt16* on hematopoietic stem cells (HSCs) specification, which are responsible for refreshing all blood and immune cells during the animal’s lifetime [31]. Based on our microarray analysis (Dataset S1), we found the expression of *wnt16* transcript was up-regulated in response to the heart injury, yet significantly repressed by beclomethasone at 1 dpa. To validate this finding, we demonstrated that *wnt16* mRNA was elevated after injury and down-regulated in zebrafish under beclomethasone treatment by RT-PCR. (Fig. 6A). We then monitored the *wnt16* mRNA expression during the cardiac repair process. The relative expression levels of *wnt16* displayed an acute increase of 10.7-fold at 1 dpa, which was reduced 2.6-fold at 3 dpa, and gradually dropped to 0.9-fold at 7 dpa (Fig. 6B). Under normal condition, the timely and transient elevation of *wnt16* implied it may be important during cardiac repair. The inhibition of *wnt16* may explain the mechanism of action on how beclomethasone hinders the cardiac repair in zebrafish.

## Discussion and Conclusion

Effective tissue repair requires precisely timed and orchestrated programming of regulated inflammatory responses in the vertebrates. As shown in this study and previous reports, pro-inflammatory cytokines work as key mediators not only for immune responses but also for additional constructive cuing effects, which initiate a series of processes including phagocyte chemotaxis, angiogenesis, and cell proliferation, all of which are critical for a competent tissue repair outcome [32], [33].

Among the known inflammatory cytokines, IL-1β and TNF-α are two early regulators of immune responses. When IL-1β and TNF-α were inhibited, the healing processes activated by other secondary cytokines responsible for subsequent constructive functions also diminished. For example, in the cutaneous wound healing-impaired glucocorticoid-treated mice, IL-1β and TNF-α expression were significantly reduced [11]. In this study, we have demonstrated that glucocorticoid treatment could impair adult zebrafish cardiac repair by interfering with conserved inflammatory responses observed in the mammals. Inhibition of early inflammatory responses in the zebrafish reduced myeloid-derived phagocyte recruitment to the cardiac injury site, followed by a down-regulation in angiogenesis and cardiomyocyte proliferation during cardiac repair.

Prevailing hypothesis has suggested that there is an intricate balance between regeneration and fibrotic healing, which are two inverse responses after injury. In higher vertebrates, the balance has been shown to lean toward fibrotic healing; and inhibition of the preceding events after injury will result in scar formation [15]. Based on the observation that mammalian fetuses with an immature immune system could heal without scarring, some studies have suggested that the immune system may play a key role in steering the repair process toward full regeneration or scarring [34]. Interestingly, in the zebrafish cardiac repair system, inhibition of the immune response led to unsuccessful regeneration, which was accompanied by excessive scar formation, suggesting the indispensable role of the immune response for completing vital organ regeneration in adult zebrafish.

Glucocorticoids are widely used to suppress various inflammatory reactions in allergic diseases, autoimmune disorders, and severe sepsis. In this study, we observed that immunosuppression using beclomethasone treatment simultaneously down-regulated angiogenic genes, including *fgfr1a* and *vegfaa* in zebrafish by 24 hours post cardiac injury. Whether the rapid inhibition of angiogenic genes was directly caused by glucocorticoid receptor activation or indirectly resulted from reduced pro-inflammatory cytokine expression and infiltrating phagocytes was not thoroughly addressed here. It is well-known that glucocorticoid receptor activation would also elevate the expression of a number of transcripts [35]; we thus do not exclude the possibility that it is theses up-regulated transcripts that are responsible for inhibiting the regenerative responses. The definite link between inflammatory responses and impaired cardiac healing needs to be further verified. Nevertheless, we demonstrate here that beclomethasone treatment would suppress neovascularization and cardiomyocyte proliferation and repression of these events may be the major causes for the detrimental effects of beclomethasone in cardiac repair. Clearly, beclomethasone treatment can create a microenvironment that no longer favors complete cardiac regeneration in zebrafish. Our results thus raise the possibility that an intervention on myocardial inflammation could provide a beneficial impact on cardiac repair that is mediated by innate immune mechanisms and may also reduce fibrotic scar formation.

In summary, our study suggests that zebrafish cardiac repair is highly associated with early immune responses. With the glucocorticoid intervention, we have demonstrated an impaired cardiac healing model can be established in adult zebrafish with a diminished regenerative capacity. This modified zebrafish model of cardiac injury could better mimic the human condition of myocardial infarction, especially on the scar removal process. The disadvantages of this approach are that the addition of anti-inflammatory chemicals would potentially generate adverse effects. Thus, researchers should design their experiments carefully when using the modified zebrafish MI model described in this study. Nevertheless, this modified model could be used to identify important factors like *wnt16* that are involved in regulating cardiac regeneration. Our findings may help to build better MI models in zebrafish that can be used to develop therapeutic interventions to enhance cardiac repair in humans, and to investigate the mechanisms that are critical in promoting functional recovery after myocardial infarction in the future.

## Materials and Methods

### Ethics Statement

The experimental procedures were approved by the committee for the use of laboratory animals at National Tsing-Hua University (IACUC number: 09808).

### Zebrafish Care

The zebrafish were maintained based on the guidelines described in the Zebrafish Book [36]. AB strain background zebrafish aged between 7 to 10 months were used for the ventricular amputation surgery. To visualize the specific cell types, the Tg(*lyz*:EGFP) and Tg(*fli1a*:EGFP) strains were used [37], [38].

### Zebrafish Ventricular Resection and Drug Delivery for Suppressing the Immune System

We followed the previously described protocol with some modifications [6]. The animals were anesthetized with a MS-222 (Sigma-Aldrich) and isoflurane (Baxter) mixture first, allowing for faster recovery to achieve a better success rate after surgery [39]. Micro-scissors were used to create a small incision on the zebrafish to quickly expose the heart. Approximately 10–20% of the ventricular apex was amputated before the fish was returned to water for recovery.

For the immunosuppression assay, adult zebrafish were pre-exposed to water containing 0.25 µM beclomethasone (Fluka), 0.25 µM ibuprofen (Sigma-Aldrich) or equivalent vehicle (i.e., 0.002% DMSO from Sigma-Aldrich, which is used to prepare the beclomethasone and ibuprofen stocks) for 1 day [13]. After receiving ventricular resection surgery, the fish were kept in isolated tanks; the fish water was changed every day for the first 3 days to avoid infection. The animals were continuously exposed to the vehicle or designated chemicals until the sacrifice.

### RNA Isolation and Real-time Quantitative Polymerase Chain Reaction (RT-qPCR)

Whole hearts were harvested and immediately homogenized in Trizol reagent (Invitrogen). We followed the standard protocol provided by the manufacturer for the remaining steps of RNA isolation. Complementary DNA (cDNA) was synthesized using SuperScript III reverse transcriptase (Invitrogen) with oligo-dT_12–18_ primers (Invitrogen) according to the protocol provided by the manufacturer. The SYBR Green master mix (ABI) was used for RT-qPCR on the ABI 7500 sequence detection system, and the data were analyzed based on the ΔΔC(t) method by ABI SDS software v1.4. [40]. β-actin mRNA expression was used as an endogenous control standard. The sequences of the primers used are provided in Table S2 For the dynamic detection of inflammatory cytokines during cardiac repair**,** three groups per time point, each composed of 6 hearts, were pooled equally to make an individual experiment; triplicate experiments were performed.

### Gene Expression Microarray and Data Analysis

Three groups of wild-type AB zebrafish, each containing 10 individuals were used in this assay. The three experimental groups are (1) no-injury (i.e. sham operation as 1dpa-vehicle, without hurting the heart), (2) 1dpa-vehicle (zebrafish were treated in 0.002% DMSO for 1 day before receiving ventricular resection. The heart was then harvested after 1 day post amputation), and (3) 1dpa-beclomethasone (zebrafish were treated with 0.25 µM beclomethasone for 1 day and underwent ventricular resection. The heart was then harvested after 1 day post amputation). After ventricular resection surgery, the three groups of test subjects were sacrificed to collect the ventricles at 1 dpa. Total RNA were extracted using Trizol reagent and eventually dissolved in reagent-grade water (Sigma-Aldrich). For the following microarray experiments, mRNA samples were sent to a specialized commercial service company, Welgene (Taiwan), for the RNA hybridization experiment; Cy3-labeled cRNA was hybridized to the Agilent zebrafish Oligo V2 4×44 k chip. The data were analyzed using GeneSpring GX (Agilent). D. rerio probes that were differentially expressed in the no-injury and 1dpa-beclomethasone groups compared to the 1dpa-vehicle group with a fold change of ≥ 3 were selected. The differentially expressed genes were clustered into separate functional groups by a corrected p value of less than 0.05 using the gene ontology analysis program in the PANTHER classification system [41]. The microarray dataset discussed in this study can be retrieved as Dataset GSE41618 from the NCBI GEO repository website (http://www.ncbi.nlm.nih.gov/geo/).

### Histology

Whole zebrafish hearts were harvested and then fixed with 2% paraformaldehyde (Merck) at 4°C overnight. The samples were sectioned with cryostat (CM3050S, Leica) at the thickness of 12 µm and stored at −80°C until use. We followed the previously described protocol with some modifications for histological staining [6]. To clearly distinguish the scar tissues from normal tissue, an acid fuchsin-orange G stain (AFOG stain) with omission of acid fuchsin step was used. Cytosol (i.e., normal tissue) should stain orange, and collagen deposition (i.e., scar tissue) should appear bright blue in the sections. For image quantification, largest 2 sections with complete morphologies at the slides were chosen to represent each sample. Acquired images were quantified with the image quantitative software, Image J (National Institutes of Health) [42].

### Bromodeoxyuridine (BrdU) Injection

For the BrdU labeling procedure, the animals were injected intraperitoneally with 10 µl of 20 mM BrdU (Sigma-Aldrich) dissolved in PBS using a Hamilton syringe (26G, Hamilton) [6]. BrdU injection was performed once a day from 6 dpa to 9 dpa; the zebrafish were then sacrificed with their hearts collected at 10 dpa.

### Immunofluorescence Staining

The harvested samples were fixed and sectioned by cryostat as mentioned above. We then followed the previously described protocol for immunofluorescence staining [6]. Primary antibodies used were mouse anti-BrdU (1∶100, Bu20a, Thermo), mouse anti-myosin heavy chain (1∶50, MF-20, DSHB), rabbit anti-myosin light polypeptide 2 (1∶600, GTX128816, GeneTex), rabbit anti-EGFP (1∶600, NB600-308, Novus), and rabbit anti-GATA4 (1∶500, ab61170, abcam). Species-specific secondary antibodies conjugated to DyLight 488 or DyLight 549 (Jackson) were used at a dilution of 1∶200. Nuclei were stained with 5 µg/ml Hoechst 33342 (Invitrogen); F-actin was stained with AlexaFluor546-phalloidin (Invitrogen) at a concentration of 1 unit/ml. Images were captured using a laser-equipped confocal microscope A1R (Nikon) or LSM 510 (Carl Zeiss). For image quantification, the largest 2 to 3 sections with complete morphologies on the slide were chosen to represent each sample; the acquired images were quantified using Image J.

### Statistics

The data were analyzed using SigmaPlot v.10 and expressed as the mean with the standard error of the mean (SEM). Statistical significance was determined by a p value <0.05 using Student’s t-test.

## Supporting Information

Figure S1 **Treatment with ibuprofen also caused impaired cardiac repair in zebrafish.** Blue: scar tissue; Orange: normal tissue. (A, A′) In the vehicle control group, the zebrafish regenerated the injured hearts perfectly 1 month after injury. (n = 3). (B, B′) Persistent exposure to ibuprofen (0.25 µM) hindered the cardiac repair process. Some scar tissue (arrow) was found in the wound instead of renewed cardiomyocytes; obvious blood clots and cellular debris were left at the wound. (arrow head) (n = 5, scale bar = 100 µm). The dashed lines indicate the approximate amputation plane.
(TIF)

Figure S2 **Beclomethasone and ibuprofen treatment efficiently repress the immune responses.** Whole hearts were harvested at 16 hours post ventricular resection. RT-qPCR was conducted to quantify the relative fold of pro-inflammatory genes. Under beclomethasone treatment, the normalized expression level of *il-1β* decreased to 3.9% , *tnf-α* decreased to 15.6%, and *ptgs-2b* decreased to 20.9% of the controls. Under ibuprofen treatment, the normalized expression level of *il-1β* decreased to 4.7% , *tnf-α* decreased to 25.2%, and *ptgs-2b* decreased to 8.8% of the controls. (n = 3) The data represent the mean± SEM, * indicates p<0.05, N.S. indicates no significant difference.
(TIF)

Figure S3 **Microarray analysis identified healing responsive D.** ***rerio*** **probes down-regulated in the impaired healing heart.** Three sets of experimental groups were used to perform the microarray hybridization experiment: no-injury, 1dpa-vehicle, and 1dpa-beclomethasone. The 1dpa-vehicle set was used as a standard to identified genes differentially expressed on fold change of ≥ 3 times. 2857 D. *rerio* probes were up-regulated after cardiac injury. In contrast, 4064 injury responsive probes were down-regulated after beclomethasone treatment. A total of 2288 probes were identified, which represent zebrafish heart transcripts that were up-regulated in response to the injury under normal condition, but significantly inhibited in zebrafish treated with beclomethasone.
(TIF)

Table S1 Gene ontology analysis of the 2288 differential expressed *D. rerio* probes.
(DOC)

Table S2 Primer list for RT-qPCR.
(DOC)

Dataset S1 **List of 2288 healing responsive D. rerio probes that were down-regulated by beclomethasone treatment.**
(XLS)
