# Supplementary figures and images for: Treatment of Glucocorticoids Inhibited Early Immune Responses and Impaired Cardiac Repair in Adult Zebrafish
Source: PLoS One. 2013 Jun 21;8(6):e66613. doi: 10.1371/journal.pone.0066613 (PMC3689762; doi:10.1371/journal.pone.0066613)

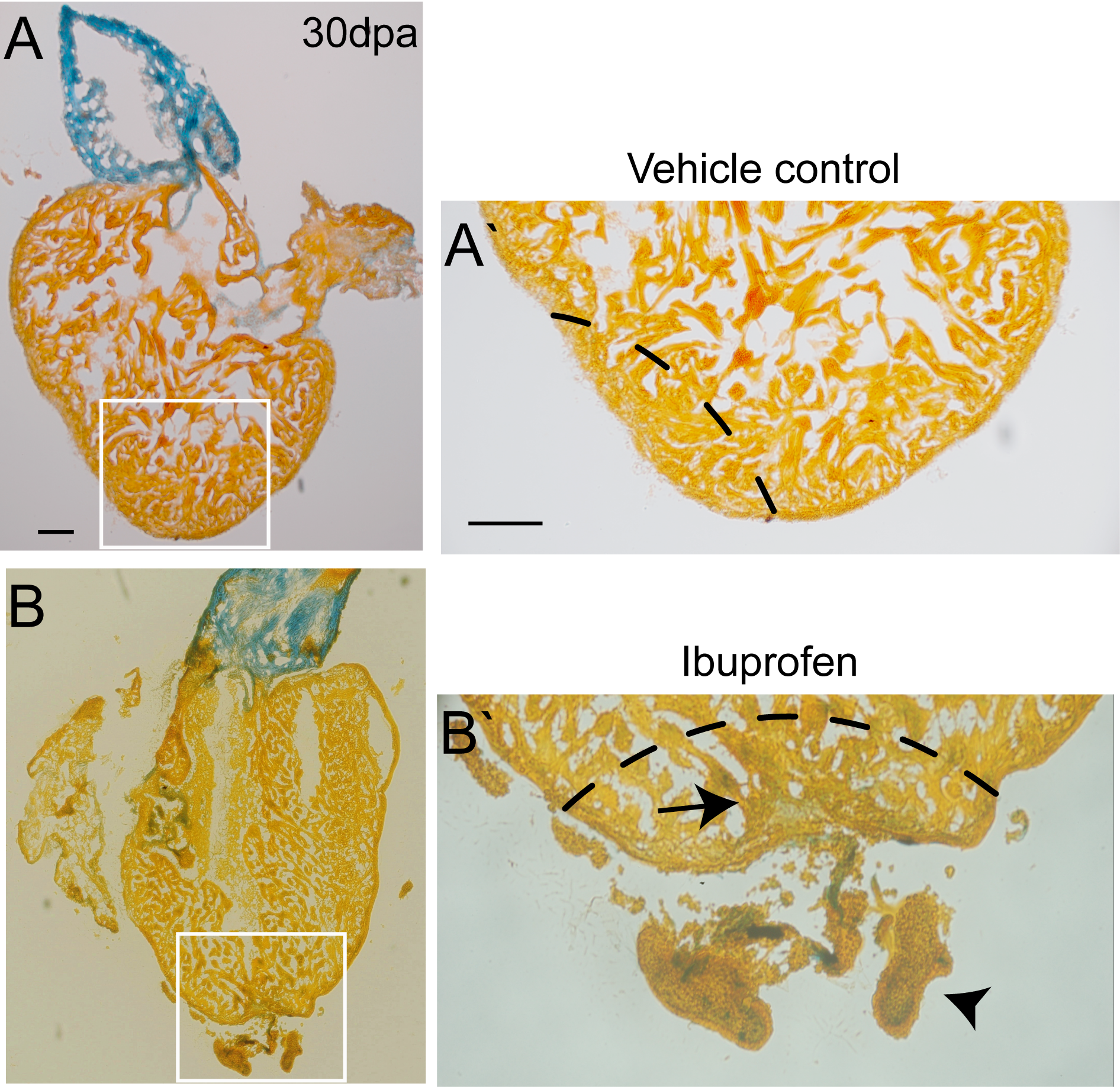

Supplement: Figure S1 — Treatment with ibuprofen also caused impaired cardiac repair in zebrafish. Blue: scar tissue; Orange: normal tissue. (A, A′) In the vehicle control group, the zebrafish regenerated the injured hearts perfectly 1 month after injury. (n = 3). (B, B′) Persistent exposure to ibuprofen (0.25 µM) hindered the cardiac repair process. Some scar tissue (arrow) was found in the wound instead of renewed cardiomyocytes; obvious blood clots and cellular debris were left at the wound. (arrow head) (n = 5, scale bar = 100 µm). The dashed lines indicate the approximate amputation plane. (TIF) [file pone.0066613.s001.tif]

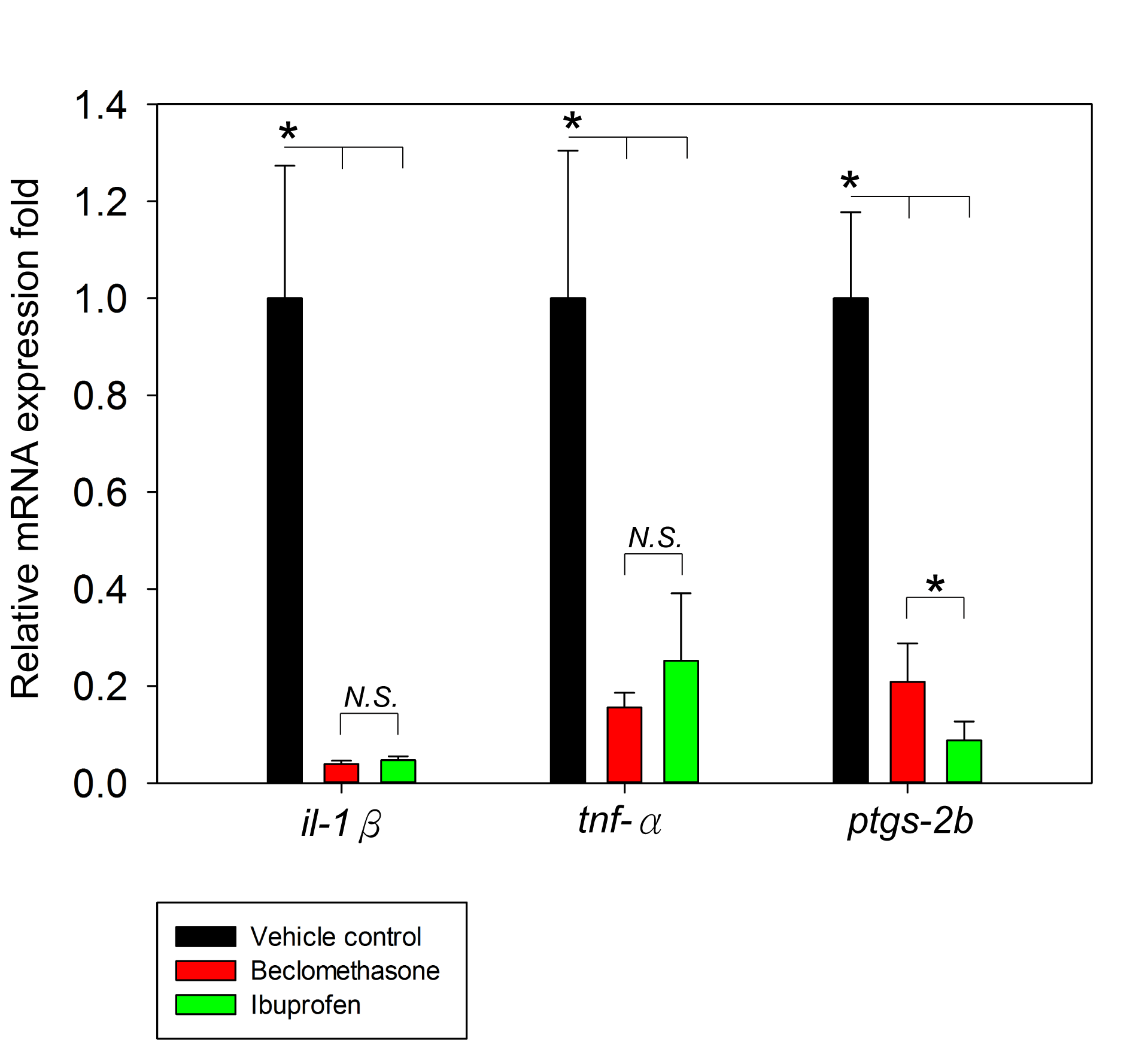

Supplement: Figure S2 — Beclomethasone and ibuprofen treatment efficiently repress the immune responses. Whole hearts were harvested at 16 hours post ventricular resection. RT-qPCR was conducted to quantify the relative fold of pro-inflammatory genes. Under beclomethasone treatment, the normalized expression level of il-1β decreased to 3.9% , tnf-α decreased to 15.6%, and ptgs-2b decreased to 20.9% of the controls. Under ibuprofen treatment, the normalized expression level of il-1β decreased to 4.7% , tnf-α decreased to 25.2%, and ptgs-2b decreased to 8.8% of the controls. (n = 3) The data represent the mean± SEM, * indicates p<0.05, N.S. indicates no significant difference. (TIF) [file pone.0066613.s002.tif]

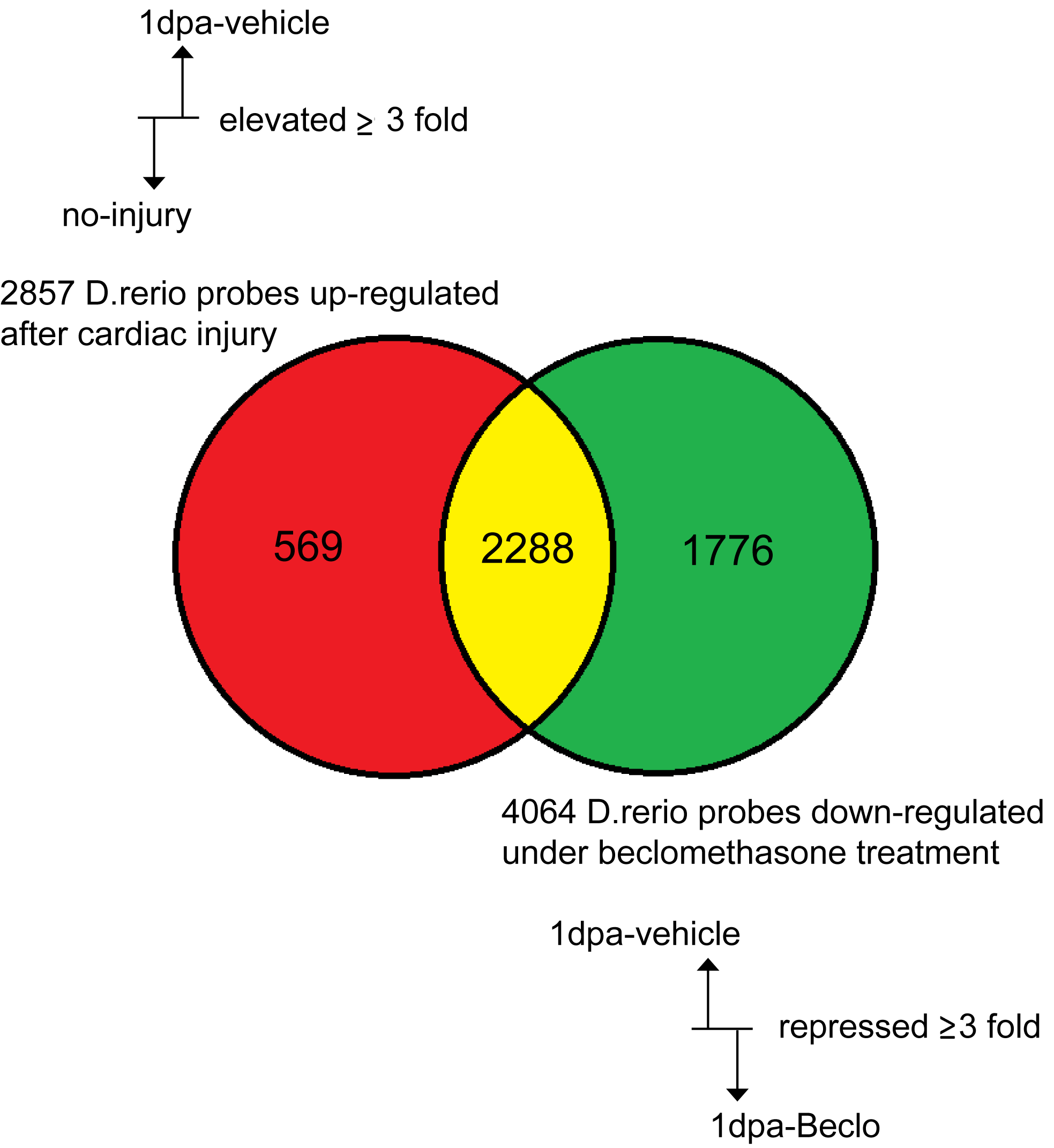

Supplement: Figure S3 — Microarray analysis identified healing responsive D. rerio probes down-regulated in the impaired healing heart. Three sets of experimental groups were used to perform the microarray hybridization experiment: no-injury, 1dpa-vehicle, and 1dpa-beclomethasone. The 1dpa-vehicle set was used as a standard to identified genes differentially expressed on fold change of ≥ 3 times. 2857 D. rerio probes were up-regulated after cardiac injury. In contrast, 4064 injury responsive probes were down-regulated after beclomethasone treatment. A total of 2288 probes were identified, which represent zebrafish heart transcripts that were up-regulated in response to the injury under normal condition, but significantly inhibited in zebrafish treated with beclomethasone. (TIF) [file pone.0066613.s003.tif]
