# Supplementary material for: Treatment of Glucocorticoids Inhibited Early Immune Responses and Impaired Cardiac Repair in Adult Zebrafish
Source: PLoS One. 2013 Jun 21;8(6):e66613. doi: 10.1371/journal.pone.0066613 (PMC3689762; doi:10.1371/journal.pone.0066613)
Supplement: Table S1 — Gene ontology analysis of the 2288 differential expressed D. rerio probes. (DOC) [file pone.0066613.s004.doc]

**Table S1**

Gene ontology analysis of the 2288 differential expressed *D. rerio* probes

| **GO Term** | **GO ID** | **P value** | **Child lineage** |
| --- | --- | --- | --- |
| RNA metabolic process | GO:0016070 | 0.0176 | RNA catabolic process |
|  |  |  | mRNA processing |
|  |  |  | mRNA transcription |
|  |  |  | rRNA metabolic process |
| system development | GO:0048731 | 0.0178 | angiogenesis |
|  |  |  | heart development |
|  |  |  | hemopoiesis |
|  |  |  | mammary gland development |
| cell communication | GO:0007154 | 0.0247 | cell-cell signaling |
|  |  |  | signal transduction |
| immune system process | GO:0002376 | 0.0249 | antigen processing and presentation |
|  |  |  | immune response |
|  |  |  | macrophage activation |
| signal transduction | GO:0007165 | 0.026 | cell surface receptor linked signal transduction |
|  |  |  | intracellular signaling cascade |
| cell surface receptor linked signal transduction | GO:0007166 | 0.027 | G-protein coupled receptor protein signaling pathway |
|  |  |  | cytokine-mediated signaling pathway |
|  |  |  | transmembrane receptor protein serine/threonine kinase signaling pathway |
|  |  |  | transmembrane receptor protein/ tyrosine kinase signaling pathway |
| mesoderm development | GO:0007498 | 0.0312 | gut mesoderm development |
| cell-cell signaling | GO:0007267 | 0.0354 | synaptic transmission |
| skeletal system development | GO:0001501 | 0.0407 | (none) |
