# Supplementary material for: Treatment of Glucocorticoids Inhibited Early Immune Responses and Impaired Cardiac Repair in Adult Zebrafish
Source: PLoS One. 2013 Jun 21;8(6):e66613. doi: 10.1371/journal.pone.0066613 (PMC3689762; doi:10.1371/journal.pone.0066613)
Supplement: Table S2 — Primer list for RT-qPCR. (DOC) [file pone.0066613.s005.doc]

**Table S2**

Primer list for RT-qPCR

| **Gene** | **Ensembl transcript ID** | **Forward primer** | **Reverse primer** | **Product Size(bp)** |
| --- | --- | --- | --- | --- |
| β-*actin* | ENSDART00000141737 | GCTGACAGGATGCAGAAGGA | TAGAAGCATTTGCGGTGGAC | 200 |
| *il-1*β | ENSDART00000015991 | TTGTGGGAGACAGACAGTGC | GATTGGGGTTTGATGTGCTT | 191 |
| *il-8* | ENSDART00000079373 | TGTTTTCCTGGCATTTCTGACC | TTTACAGTGTGGGCTTGGAGGG | 151 |
| *tnf-*α | ENSDART00000025847 | ACAAGGCAATTTCACTTCCA | AGCTGATGTGCAAAGACACC | 194 |
| *ptgs-2b* | ENSDART00000010028 | GGAGCTTTATGCTGGTTTGC | ACATGGCCCGTTGACATTAT | 226 |
| *mpx* | ENSDART00000043961 | TCGTAGTTTGGGCTTTGTGA | TAGCCTCAAACGGGAAAAAG | 213 |
| *lipocortin-1* | ENSDART00000014034 | CGGCCATTCAGAAAGAAACA | CAGTGCGCACAATGACTTTT | 186 |
| *fgfr1a* | ENSDART00000074774 | CTCCCGAAGCTCTGTTTGAC | GCCAACAATCCCTCATCATC | 210 |
| *vegfaa* | ENSDART00000128775 | TTGTGTAGTTCGCTGCACCT | CACTTCTCCACCGAGCTGAT | 203 |
| *pcna* | ENSDART00000076304 | CCCGATTGTGACCCTCTAAA | TTGGAATGAGCAGTTGGACA | 216 |
| *nkx2.5* | ENSDART00000022377 | GCATCAGAGCTTGGTGAACA | ATGCGCACGCATAAACATTA | 172 |
| *wnt16* | ENSDART00000132453 | AATCGCCCAATTATTGCTTG | GCGAACATAACAGCACCAGA | 188 |
